# Filtration based assessment of CTCs and CellSearch® based assessment are both powerful predictors of prognosis for metastatic breast cancer patients

**DOI:** 10.1186/s12885-018-4115-1

**Published:** 2018-02-20

**Authors:** Hanna Huebner, Peter A. Fasching, Walter Gumbrecht, Sebastian Jud, Claudia Rauh, Mark Matzas, Peter Paulicka, Katja Friedrich, Michael P. Lux, Bernhard Volz, Paul Gass, Lothar Häberle, Franziska Meier-Stiegen, Andreas Hartkopf, Hans Neubauer, Katrin Almstedt, Matthias W. Beckmann, Tanja N. Fehm, Matthias Ruebner

**Affiliations:** 1 Department of Gynecology and Obstetrics, Comprehensive Cancer Center Erlangen-EMN, University Hospital Erlangen, Friedrich-Alexander University of Erlangen-Nuremberg, Universitaetsstraße 21-23, 91054 Erlangen, Germany; 2 Siemens Healthcare GmbH, Günther-Scharowsky-Str.1, 91058 Erlangen, Germany; 3 Biostatistics Unit. Department of Gynecology and Obstetrics, Comprehensive Cancer Center Erlangen-EMN, University Hospital Erlangen, Friedrich-Alexander University of Erlangen-Nuremberg, Universitaetsstraße 21-23, 91054 Erlangen, Germany; 4 Department of Gynecology and Obstetrics, Heinrich Heine University of Düsseldorf, Moorenstr. 5, 40225 Duesseldorf, Germany; 5 Department of Gynecology and Obstetrics, University Hospital Tuebingen, Calwerstraße 7, 72076 Tuebingen, Germany; 6 Department of Obstetrics and Gynecology, Johannes Gutenberg University, Langenbeckstrasse 1, 55131 Mainz, Germany

**Keywords:** CTC, CellSearch, Breast cancer, Overall survival, Filtration

## Abstract

**Background:**

The assessment of circulating tumor cells (CTCs) has been shown to enable monitoring of treatment response and early detection of metastatic breast cancer (MBC) recurrence. The aim of this study was to compare a well-established CTC detection method based on immunomagnetic isolation with a new, filtration-based platform.

**Methods:**

In this prospective study, two 7.5 ml blood draws were obtained from 60 MBC patients and CTC enumeration was assessed using both the CellSearch® and the newly developed filtration-based platform. We analyzed the correlation of CTC-positivity between both methods and their ability to predict prognosis. Overall survival (OS) was calculated and Kaplan-Meier curves were estimated with thresholds of ≥1 and ≥5 detected CTCs.

**Results:**

The CTC positivity rate of the CellSearch® system was 56.7% and of the filtration-based platform 66.7%. There was a high correlation of CTC enumeration obtained with both methods. The OS for patients without detected CTCs, regardless of the method used, was significantly higher compared to patients with one or more CTCs (*p* < 0.001). The median OS of patients with no CTCs vs. ≥ 1 CTC assessed by CellSearch® was 1.83 years (95% CI: 1.63–2.02) vs. 0.74 years (95% CI: 0.51–1.52). If CTCs were detected by the filtration-based method the median OS times were 1.88 years (95% CI: 1.74–2.03) vs. 0.59 years (95% CI: 0.38–0.80).

**Conclusions:**

The newly established EpCAM independently filtration-based system is a suitable method to determine CTC counts for MBC patients. Our study confirms CTCs as being strong predictors of prognosis in our population of MBC patients.

## Background

Breast cancer is the most common cancer in women, with one out of eight women developing this type of cancer during life [1]. Even though the therapeutic management has significantly improved during the last decades, especially metastatic breast cancer (MBC) is still an incurable disease with a 5-year survival rate of less than 25%. This long term outcome for MBC is influenced by various biological factors. Tumor characteristics, which are associated with breast-cancer related deaths, like blood-derived metastatic potential and the presence of micrometastases are difficult to assess by classical morphological imaging techniques. Within the last years, liquid biopsy procedures for gaining prognostic information associated with the possibility of metastasis formation were developed [2–4]. Circulating tumor cells (CTCs) are potential founder cells for metastasis and can be collected and enriched from patients’ blood samples. Their numeration has been proven to be of highly prognostic impact [5] and furthermore allows physicians to recommend a personalized therapy and to monitor treatment response.

Different methods for the assessment of CTCs have been described so far. Most of them rely on the identification of CTCs by targeting antigens specific for epithelial cells (e.g. epithelial cell adhesion molecule, EpCAM) [6], by physical characteristics [7, 8] or expression patterns [9, 10]. The gold standard for CTC counting is the FDA approved semi-automated CellSearch® system (Veridex, LLC, Warren, NJ, USA). This technique uses an immunomagnetic selection of EpCAM-positive CTCs followed by immunostaining of cytokeratins (CKs) and CD45 [11]. So far, many studies presented a significant correlation of the CTC count assessed by CellSearch® (CTC_CS_) and the progression-free as well as the overall survival of MBC patients [12–15]. Thus, CellSearch® represents a platform of high impact to analyze the prognosis and treatment response of breast cancer patients. However, limitations of this method are the missing detection of EpCam-negative CTCs and the difficulties in adding downstream applications like RNA, DNA or protein analysis of captured CTCs.

In this study we aimed to compare the established CellSearch® system with a new, filtration-based method on an integrated CTC platform for automated cellular protein and nucleic acid analysis. Overall, we focused on the comparability of both units and the prognostic value for MBC patients.

## Methods

### Study design and patient characteristics

CTC analysis was performed for a total of 60 MBC patients enrolled in the iMode-B (imaging and molecular detection breast) study. Patients were included between 2010 and 2012 at the University Breast Center Franconia, Erlangen. Inclusion criteria were radiologically measureable or clinically assessable MBC and a written informed consent given by the patients for the use of their blood samples. The study was approved by the ethics committee of the Medical Faculty, Friedrich-Alexander University Erlangen-Nuremberg. There were no exclusion criteria based on tumor subtype, age or other patient characteristics. Physicians were blinded to CTC test results and investigators performing CTC analysis were blinded to the clinical data.

### Data capturing

Data was documented in an electronic case report form specialized on the documentation of MBC by trained and dedicated staff. The database had the same structure like the PRAEGNANT study [16, 17] and data are monitored using automated plausibility checks. The documented data comprised information about primary diagnosis, surgery, treatment as well as progression and information about death. Histopathological data from the primary tumor were documented from pathology reports. Patients were considered estrogene receptor (ER) or progesterone receptor (PR) positive if by immunohistological (IHC) staining at least 1% of cells were stained positive. HER2 positivity was defined as either having an IHC score of 3+ or a gene amplification as shown by chromogenic in situ hybridization.

### Circulating tumor cell detection with the CellSearch® system (CTC_CS_)

Blood samples were drawn into CellSave Tubes (Veridex, LLC) and shipped overnight to an experienced and dedicated laboratory (T.N.F). The CellSearch® Epithelial Cell Test (Veridex, LLC) was applied for CTC enrichment and enumeration as described before [6, 10, 18, 19]. In brief, CTCs were captured with the automated CELLTRACKS® AUTOPREP® System by using anti-EpCAM-antibody bearing ferrofluid followed by their detection with immunostaining of CKs 8, 18 and 19 and the leukocyte common antigen CD45 as well as 4′,6-diamidino-2-phenylindole (DAPI) to ensure integrity of the cell nucleus. CTCs were identified and enumerated by automated fluorescence microscopy using the CELLTRACKS ANALYZER II® System.

### Circulating tumor cell detection with the filtration based system (CTC_FB_)

For the filtration based method blood samples (7.5 ml EDTA-blood) were processed with the modified pipetting robot of the VERSANT® kPCR Sample Prep system (SIEMENS Healthcare GmbH, Erlangen). Up to 8 samples could be processed in parallel. For that purpose, 50 ml Falcon tubes, each filled with 22.5 ml red blood cell- (RBC-) lysis buffer (1.5 M NH_4_Cl, 100 mM NaHCO_3_, 10 mM disodium EDTA in Millipore water) were placed into a rack of the pipetting robot. The 7.5 ml EDTA blood samples were transferred into individual falcon-tubes by the robot and incubated at RT for 15 min by back and forth aspiration of the pipettes. Subsequently the RBC-lysed diluted blood samples were pipetted into individual vacuum-based filtration units (Siemens Healthcare). CTCs were selected by cell size using Whatman nuclepore track etched membranes (GE) with a defined pore size of 8 μm and 1 inch diameter. This filter system, in combination with dedicated filtration-pressure control (10–30 mbar negative pressure) enables the retention of 85–100% of tumor cells with a background of approx. 0.1% remaining white blood cells. After cell capture and fixation by 3.6% Formaldehyde (Sigma Aldrich) in PBS, the cells were washes and the membrane was permeabilized using Triton X100 (Fluka). In order to perform automated immunostaining, non-specific binding sites were blocked using Blocking Solution (Candor) and cells were stained for cytokeratin 8, 18 (5 μg/ml mouse anti-CK8/18-Dy550, clone UCD/PR 10.11, Siemens Healthcare) and cytokeratin 19 (5 μg/ml mouse anti-CK19-Dy550, clone A53-B/A2, Siemens Healthcare), CD45 (20 μg/ml mouse anti-CD45-Dy650, clone 9.4, Siemens Healthcare) and DAPI (1.1 μg/ml, 4′,6-Diamidino-2-phenylindole dihydrochloride, Sigma Aldrich) by pipetting corresponding antibody-fluorophore-conjugate solutions together with DAPI for cell nucleus staining. Cover medium (1,4-Diazabicyclo [2.2.2] octane, DABCO, Sigma Aldrich) was added to preserve the fluorescence intensity. Finally, the filtration membranes were removed from the processing robot for optical investigation. Cytokeratin positive/CD45 negative/DAPI positive cells (CTCs) were counted by fluorescence scanning microscopy using a dedicated software solution (SIEMENS Healthcare GmbH).

### Statistical analysis

CTC assessments were described with cross tables using two different cut-offs (0 vs. ≥1) [20] and (< 5 vs. ≥5) [10]. CTC positivity with regard to prognostic value was defined as detecting at least one CTC in the blood samples with the respective method for CTC detection. A Wilcoxon signed-rank test was performed to compare CTC counts assessed by the different detection methods. A significant test result indicates that there are systematic differences between both detection methods. Furthermore, Spearman’s rank correlation coefficient was calculated.

Overall survival was defined as the elapse time between the blood draw and the time of death or last follow-up, if no death event occurred during observation time. The maximal observation time of a patient was approximately 5 years. Survival rates were estimated using the Kaplan-Meier product limit method. Confidence interval of median survival time was estimated as described in [21]. Survival rates of patients with or without CTCs were compared using the log-rank test. Cox proportional hazards models were used to investigate the prognostic value of each CTCs assessment (one model for CTC_CS_ and one model for CTC_FB_) in addition to other known prognostic factors [22]. Those prognostic factors were age at diagnosis (continuous), hormone receptor and HER2 status (positive vs. negative), grading (ordinal), therapy line (ordinal).

All tests were two-sided, and a *p*-value of ≤0.05 was regarded as statistically significant. Calculations were carried out using the software package SPSS (Version 21, IBM).

## Results

### Patient and tumor characteristics

The patient population consisted of 60 patients with a mean age of 60.9 years (SD, 11.2). A total of 16 patients were treated with first-line therapy, 12 with second line-therapy, 12 with third-line therapy and 18 with higher therapy-lines than third line (Table 1). A total of 27 patients received a chemotherapy at time of blood draw, 18 were treated with an antihormon treatment (AH) at time of blood draw and 42 patients were treated with a therapy other than the standard AH or chemotherapy. Of all 60 patients 70.0% had an ER, 63.3% PR and 20.0% HER2 positive tumor (Table 1). Further detailed patient characteristics are shown in Table 1.

**Table 1:** Patient characteristics

Group			
Age, years (mean, SD)		60.9	11.2
Body mass index (mean, SD)		26.9	6.1
Tumor stage (n, %)	T1	18	30.0
	T2–4	40	66.6
	n.a.	2	3.3
N-staging (n, %)	N+	36	60.0
	N0	18	30.0
	n.a.	6	10.0
ER (n, %)	Positive	42	70.0
	Negative	17	28.3
	n.a.	1	1.7
PR (n, %)	Positive	38	63.3
	Negative	21	35.0
	n.a.	1	1.7
HER2 (n, %)	Positive	12	20.0
	Negative	41	68.3
	n.a.	7	11.7
Grading (n, %)	G1	4	6.7
	G2	27	45.0
	G3	28	46.7
	n.a.	1	1.7
Histopathological subtype (n, %)	Ductal	50	83.3
	Lobular	7	11.7
	Others	2	3.4
	n.a.	1	1.7
Treatment line^a^ (n, %)	First line	16	26.7
	Second line	12	20.0
	Third line	12	20.0
	Higher	18	30.0
	n.a.	2	3.3
Treatment at blood draw (n, %)	Chemotherapy	27	45.0
	AH	18	30.0
	Others^b^	42	70.0

^a^Therapy lines are either chemotherapies, antihormone therapies or other anti-cancer treatments. Each initiated therapy line is counted as one regardless of whether a disease progression triggered the therapy initiation

^b^e.g. bone modifying drugs or monoclonal antibodies

### CTC results

At least one CTC was found in 66.7% (*n* = 40) of the patients with the filtration method and in 56.7% (*n* = 34) with the CellSearch® method. There were 4 cases which were CTC positive according to the CellSearch® method, but CTC negative using the filtration method. Vice versa, in 10 cases the filtration method detected CTCs and the CellSearch® method did not. Overall accuracy rates comparing positive vs. negative test results was 76.7% (*n* = 46). Considering a classification with CTC negative vs. 1–4 CTCs vs. ≥5 CTCs, the overall accuracy rate was 60% (*n* = 36) (Table 2).

**Table 2:** Comparison of CTC enumeration by CellSearch® and filtration based method

		CTC_FB_ n (%)			
		Negative	1–4 CTCs	≥ 5 CTCs	Total
CTC_CS_ n (%)	Negative	16 (26.7%)	8 (13.3%)	2 (3.3%)	26 (43.3%)
	1–4 CTCs	3 (5.0%)	5 (8.3%)	10 (16.7%)	18 (30.0%)
	≥ 5 CTCs	1 (1.7%)	0 (0%)	15 (25.0%)	16 (26.7%)
	Total	20 (33.3%)	13 (21.7%)	27 (45.0%)	60 (100%)

**Fig. 1:**
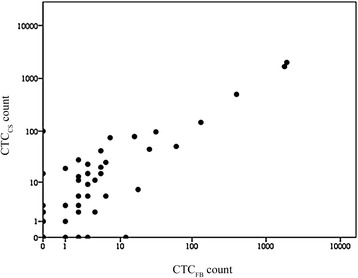
Correlation of CTC_CS_ and CTC_FB_ counts

Comparing the CTC counts assessed by CellSearch® method and the filtration based system of each patient, we found a high correlation (Spearman’s correlation 0.733) of the CTC enumeration (Fig. 1). The CellSearch® system detected a range of 1 to 2000 CTCs while the filtration based method counted CTCs from 1 to 1900. Overall the CTC enumeration by CellSearch® (median: 2.5 cells) was slightly higher compared to the one assessed with the filtration method (median: 1.5 cells). The cell count was lower with the filtration method in 33 cases and higher in 9 cases, a tie was seen in 18 cases of which 16 were a pair of 0 and 0 counts (*p* < 0.001, Wilcoxon test).

**Fig. 2:**
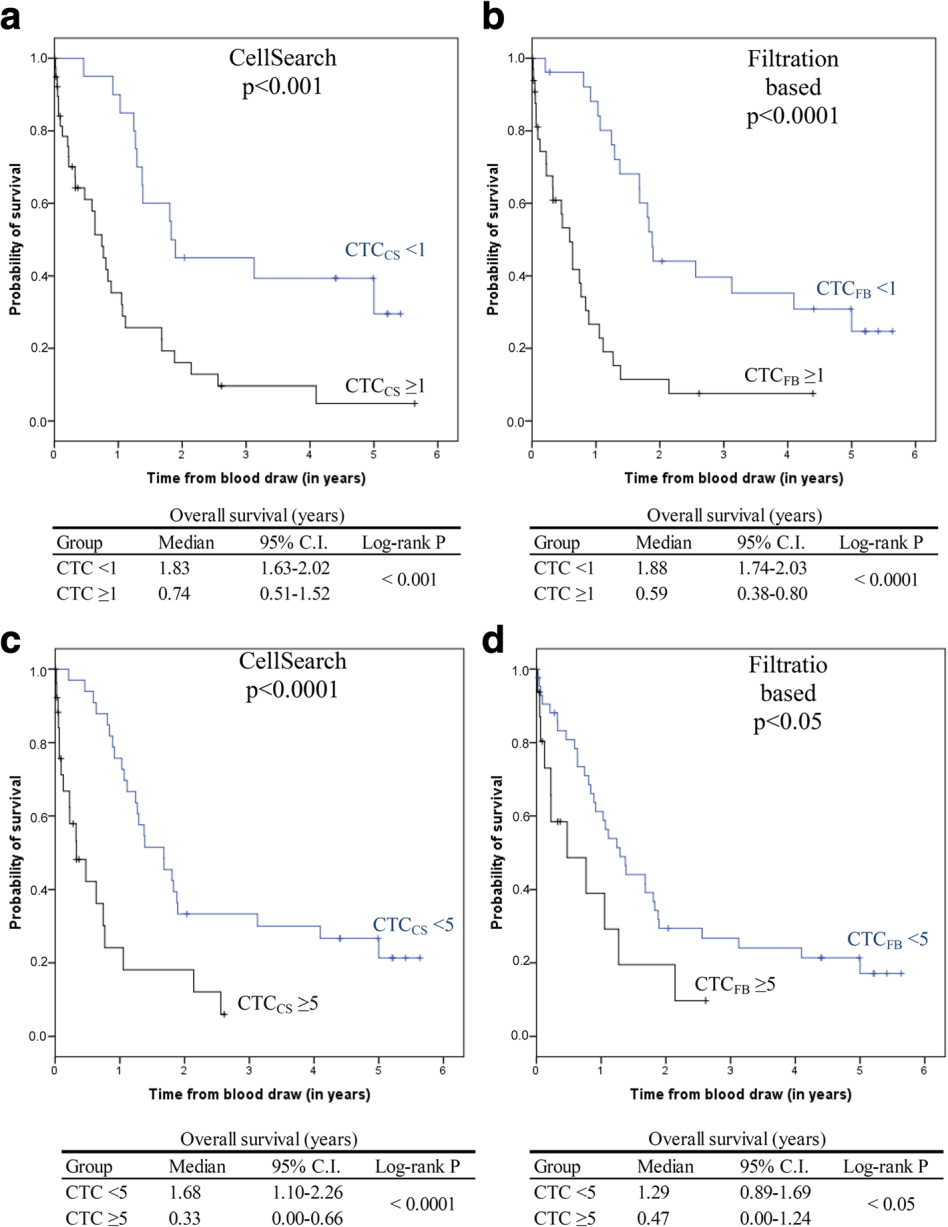
Overall survival with regard to CTC_CS_ (**a** threshold ≥1 and **c** threshold ≥5) and CTC_FB_ count (**b** threshold ≥1 and **d** threshold ≥5)

CTC significantly influenced overall survival in addition to the considered predictors. The adjusted hazard ratio (HR) for CTC_CS_ was 5.2 (95% CI: 2.2–12.4) and for CTC_FB_ the HR was 4.2 (95% CI: 1.9–9.4). The results of both Cox models are shown in Table 3 and Table 4. Kaplan-Meier curves for overall survival grouped into positive or negative CTC count assessed by CellSearch® or the filtration based method are shown in Fig. 2a and Fig. 2b. Kaplan-Meier curves with a threshold of ≥5 CTCs are displayed in Fig. 2c and Fig. 2d.

**Table 3:** Cox Regression model for the prediction of OS using CTC count by CellSearch® and covariates

Characteristic		HR	95% CI	*p*-value
Age	Per year	1.00	0.97–1.03	0.91
Hormone receptor status	Negative	1 (reference)		
	Positive	0.44	0.17–0.85	0.08
HER2 Status	Negative	1 (reference)		
	Positive	0.32	0.13–0.85	0.02
Grading	Per grade	1.08	0.58–2.02	0.82
Therapy line	Per line	1.01	0.78–1.32	0.93
CTC count	0	1 (reference)		
	≥1	5.20	2.18–12.43	0.0002

**Table 4:** Cox Regression model for the prediction of OS using CTC count by the filtration based method and covariates

Characteristic		HR	95% CI	*p*-value
Age	Per year	0.99	0.96–1.02	0.47
Hormone receptor status	Negative	1 (reference)		
	Positive	1.50	0.56–4.06	0.41
HER2 Status	Negative	1 (reference)		
	Positive	0.89	0.35–2.25	0.80
Grading	Per grade	1.45	0.70–3.00	0.32
Therapy line	Per line	0.98	0.75–1.26	0.85
CTC count	0	1 (reference)		
	≥1	4.20	1.86–9.46	0.001

The median overall survival of 1.83 years (95% CI: 1.63–2.02) for patients with < 1 CTC_CS_ (Fig. 2a) was similar to the median overall survival of 1.88 years (95% CI: 1.74–2.03) for patients with no CTC_FB_ count (Fig. 2b). In contrast the median overall survival of 0.59 years (95% CI: 0.38–0.80) for patients with 1 or more CTCs assessed by the filtration based method (Fig. 2b) was slightly shorter compared to the overall survival of 0.74 years (95% CI: 0.51–1.52) for patients with ≥1 CTC_CS_ (Fig. 2a). Similarly, significant differences regarding the overall survival were detected for both CTC_CS_ and CTC_FB_ counts with a threshold of ≥5 CTCs (Fig. 2c and Fig. 2d). The median overall survival of 1.68 years (95% CI: 1.10–2.26) for patients with < 5 CTC_CS_ (Fig. 2c) was slightly longer than the median overall survival of 1.29 years (98% CI: 0.89–1.69) for patients with < 5 CTC_FB_ counts (Fig. 2d). In comparison, the median overall survival of 0.33 years (95% CI: 0.00–0.66) for patients with ≥5 CTC_CS_ (Fig. 2c) was similar to the median overall survival of 0.47 years (95% CI: 0.00–1.24) for patients with 5 or more CTCs assessed by the filtration based method (Fig. 2d).

## Discussion

In this study we used CellSearch®, a commonly used method for CTC detection, and a new established automated filtration-based method to assess the prognostic value of CTC count in peripheral blood in a cohort of MBC patients. The CTC count within 7.5 ml of blood draw was determined in a study cohort of 60 radiologically measureable or clinically assessable MBC patients. We calculated the overall survival to determine and compare the prognostic impact of both methods. Even though the most commonly used cutoff for CTC positivity is five or more, it is still unclear whether a presence of one or more CTCs might be an even more accurate predictor depending on the tumor subgroup analyzed [20, 23]. Several prospective, multicenter studies showed a significant prognostic value for progression-free and overall survival of MBC patients with CTC levels < 5 or < 1 [12, 14, 24]. Additionally, CTC assessment was proven to be a good setting for valuation of treatment response and as an individual predictive test for metastatic relapse [14, 24, 25]. Here, we set out to compare both thresholds (≥1 and ≥5 CTCs) for both methods. A significant prognostic value of CTC count could be achieved using the CellSearch® as well as the filtration-based system. There were no differences between a threshold of one CTC or five CTCs, indicating that the new filtration-based method is also suitable for sensitive detection of less than five CTCs.

Probably the most discussed downside of the CellSearch® method is that the CTC detection and isolation relies only on EpCAM positivity [26]. It is known that tumor cells and in particular CTCs are highly heterogeneous and are able to change their expression profiles during cancer growth and spreading [27]. Especially during epithelial to mesenchymal transition epithelial surface molecules get lost to allow detachment and invasion of tumor cells, while these markers are re-acquired during mesenchymal to epithelial transition [26, 28]. During cancer cell dissemination the epithelial surface marker EpCAM can be downregulated by either DNA methylation, glycosylation or proteolytic cleavage allowing the cancer cells to switch to a more mesenchymal and invasive phenotype [29, 30]. This emphasizes that a method only relying on EpCAM positivity may not be suitable for detection of all CTCs and thus might give inadequate results concerning the prognostic value or the biological classification the CTCs. In contrast to the CellSearch® system, the filtration-based system does not select CTCs based on EpCAM positivity and thus we hypothesize this system might be suitable for detection of CTCs with a wider range of different phenotypes. We assume the detection of patients with a positive CTC_FB_ count but negative CTC_CS_ enumeration might be due to the missing EpCAM positivity of these cells.

Overall, the assessment of the filtration based method was feasible. The CTC positivity was within the expected rate and similar to results from different studies [6, 10]. Interestingly, even though the filtration based method does not only select EpCam positive CTCs but in contrast to the CellSearch® system also EpCAM negative ones, we observed an overall smaller CTC count with the filtration based system compared to CellSearch®. Nevertheless, we could show a significant prognostic value for overall survival using both methods. We hypothesize that the smaller number of detected CTCs might be due to the defined pore size of the filtration based system. It was shown earlier that CTCs from prostate cancer patients, which were isolated using the CellSearch® system, had a significant smaller average diameter (7.97 μm) compared to cultured prostate cancer cells [31]. Even though, to our knowledge, there are no studies regarding the tumor cell size of CTCs from breast cancer patients collected by the CellSearch® system, we assume similar findings would occur. Our filtration based system only collects CTCs with a diameter of 8.0 μm or larger and thus, this might be causative for the overall smaller cell numbers and the CTC_CS_ positive, but CTC_FB_ negative enumerations.

The assessment of tumor characteristics on CTCs is an attractive opportunity to avoid repeated tissue biopsies. CTC counts from peripheral blood samples are defined as liquid biopsies. In contrast to tissue biopsies, this is a non-invasive, quick and feasible real-time method to gain tumor cells for further analysis. Tumor characteristics can help to stratify therapy decisions. Even if primary tumor biopsies are negative for certain tumor markers (e.g. HER2), CTCs often show a different expression pattern (HER2 positive) [23]. These characteristics of CTCs are important hallmarks to define the treatment strategy and can help to avoid overtreatment. The ongoing DETECT III trial (NCT01619111) is currently investigating the therapeutic relevance of HER2-targeted therapy for MBC patients with HER2-negative tissue biopsies but HER2-positive CTCs [32]. Additionally, protein or gene expression profiles and analysis of epigenetic or genetic alterations of the DNA could help to characterize CTCs and thus the tumor even further [33, 34]. It even might help to stratify the therapeutic strategy for MBC patients [35]. As the filtration-based setting for CTC isolation is based on an automated nucleic acid preparation system (VERSANT® kPCR sample Prep system), it might not only help to determine the CTC count but also to purify DNA, RNA or proteins from CTCs for further analysis [36].

Nevertheless, this study has several limitations. First, the small sample size only allows to coarsely compare the two methods with regard to their prognostic value. Second, the lack of standardized treatment is a potential bias as it might influence the prognostic value regardless of the CTC count. Additionally, the time of blood draw was not defined precisely. But overall this setting represents the common clinical practice and was sufficient enough to compare two different CTC detection techniques in regard of overall survival.

## Conclusions

In summary, our data indicates that the newly established EpCAM independently filtration-based method might be equivalent to the CellSearch® method in regard to sensitivity of detecting CTCs from MBC patients and predicting prognosis. The filtration-based method might be easier to be used for automated RNA, DNA or Protein extraction from isolated CTCs allowing an in-depth characterization of the CTCs and the related biological background of the metastatic disease.

## Abbreviation

CTC: Circulating tumor cells
CTC_CS_: CTCs assessed using CellSearch® method
CTC_FB_: CTCs assessed using the filtration-based method
ER: Estrogene receptor
IHC: Immunohistological
MBC: Metastatic breast cancer
PR: Progesterone receptor
